# Breast Implant-Associated Anaplastic Large Cell Lymphoma Presenting in a Postpartum Patient: A Case Report

**DOI:** 10.7759/cureus.43334

**Published:** 2023-08-11

**Authors:** Seema A Al-Shaikhli, Starr Mautner, Niloofar Nasseri-Nik, Katharine Lampen-Sachar

**Affiliations:** 1 Radiology, Herbert Wertheim College of Medicine at Florida International University, Miami, USA; 2 Surgery, Baptist Health South Florida, Miami, USA; 3 Pathology, Baptist Health South Florida, Miami, USA; 4 Breast Imaging, Baptist Health South Florida, Miami, USA

**Keywords:** breast implant-associated anaplastic large cell lymphoma, breast lymphoma, general surgery breast cancer, breast cancer imaging, breast oncology, mri breast

## Abstract

Breast implant-associated anaplastic large cell lymphoma (BIA-ALCL) is a rare cancer found in women with breast implants, occurring approximately eight years after their placement. Since the initial case report of BIA-ALCL in 1997, over 700 cases have been described worldwide and there is now a registry of cases diagnosed in the United States to help learn more about this disease process and prognosis. The majority of cases have been associated with Allergan’s textured implants which have been recalled. As this disease is relatively rare with only 1,130 cases described worldwide, it is important to keep BIA-ALCL in mind in the differential diagnosis of a woman presenting with breast swelling and peri-implant fluid collection in the setting of having a textured implant. Outcomes for women with BIA-ALCL are favorable, as most disease is contained within the peri-implant capsule. Thus, complete surgical resection is often curative. Here, we report the case of a 40-year-old woman with BIA-ALCL who presented with unilateral breast swelling in the peripartum period.

## Introduction

Breast augmentation remains one of the most common cosmetic procedures in the United States, with approximately 365,000 procedures performed in 2021 [1]. Textured breast implants gained popularity in the 1980s as data released during this time revealed lower capsular contracture rates, which, in turn, would provide a more natural and aesthetically desired breast shape [2]. This benefit was theorized to be due to the increased surface area of the implant prolonging the process of inflammation, thus preventing a coordinated response by fibroblasts to lay a strong collagen matrix [2]. The textured exterior was also believed to provide better adherence to host tissue, preventing malrotation and distortion of the implant within the chest [3]. However, associations between textured breast implants and anaplastic large cell lymphoma were first reported in 1997 and, subsequently, gained more attention in 2011 after the release of a safety communication by the Food and Drug Administration (FDA) [3]. By 2019, the FDA issued a voluntary recall of Allergan’s textured breast implants, after investigations revealed the association of breast implant-associated anaplastic large cell lymphoma (BIA-ALCL) with textured breast implants, primarily with Allergan’s product [4]. Soon after, Allergan issued a global recall of its product [5].

Patients with BIA-ALCL initially present with late-onset breast swelling, asymmetry, or pain several years after breast implant placement [6]. Clinical examination of the breast reveals a peri-implant seroma in most cases, with an associated mass in 15% of patients [6]. Ultrasound is the first-line imaging modality recommended to evaluate for the presence of a seroma with a possible mass [6]. MRI is used to further characterize the seroma and identify peri-implant solid components [6].

Few case reports include the presentation of BIA-ALCL in a postpartum patient, such as ours, which is important for highlighting a possible differential diagnosis of a fluid collection in the breast of a postpartum woman. Our case report provides a comprehensive review of the classic radiological and pathological findings seen in BIA-ALCL.

## Case presentation

A 40-year-old postpartum patient presented one month after giving birth with two months of unilateral left breast swelling and associated shooting pains in the breast. The patient initially believed that she had mastitis, but even after cessation of breastfeeding shortly after giving birth, her symptoms persisted and she progressed to develop night sweats. Her physical examination revealed left breast enlargement and tenderness with a large pericapsular fluid collection and a soft, mobile left axillary lymph node. There were otherwise no dominant masses in either breast, no skin changes, and no nipple discharge. Well-healed periareolar scars were noted due to the patient’s initial breast augmentation and subsequent elective breast implant exchange seven years prior. The patient had in place textured breast implants.

Her only significant past medical history was human papillomavirus. She had no personal history of cancer but did have a family history notable for breast cancer in her paternal grandmother and ovarian cancer in her maternal grandmother.

**Figure 1:**
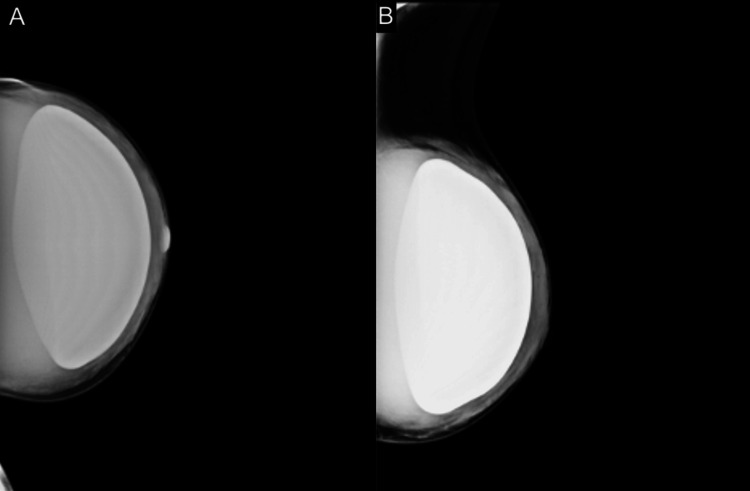
(A) Craniocaudal and (B) mediolateral oblique views of the left breast demonstrating increased parenchymal density surrounding a pre-pectoral silicone implant.

**Figure 2:**
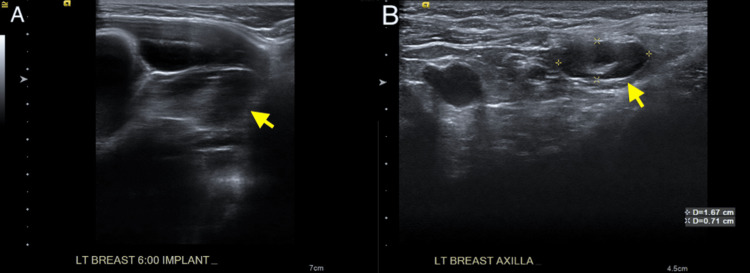
(A) Static grayscale ultrasound image of the left breast 6:00 demonstrating a complex fluid collection with internal septations surrounding the partially imaged silicone implant. (B) Static grayscale ultrasound image of the left axilla demonstrating two morphologically abnormal-appearing axillary lymph nodes with associated cortical thickening and increased size.

**Figure 3:**
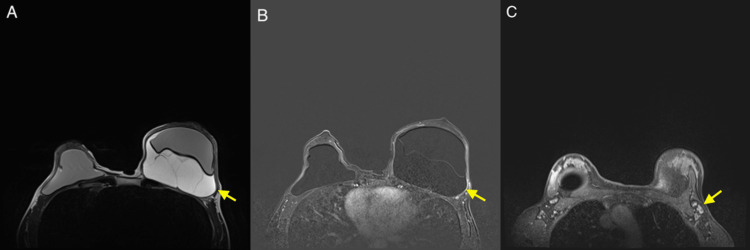
(A) Axial T2-weighted MRI of the breasts demonstrating a complex T2 hyperintense fluid collection with internal septations surrounding the left breast pre-pectoral silicone implant, displacing the implant anteriorly. (B) Axial T1 post-contrast with fat saturation image demonstrating only minimal peripheral enhancement along the lateral aspect of the peri-implant collection capsule wall. (C) Axial T1 post-contrast with fat saturation image demonstrating asymmetrically prominent left axillary lymph node with associated cortical thickening.

The patient underwent a bilateral breast diagnostic mammogram (Figure 1) and ultrasound (Figures 2A, 2B) which revealed a large peri-implant fluid collection with septations in the left breast and no definite solid component. A prominent lymph node in the left axilla with cortical thickening was present, with cortical thickness of up to 4 mm. The patient subsequently had a breast MRI with and without contrast which re-demonstrated the large peri-implant fluid collection with internal septations, mild associated peripheral enhancement, as well as asymmetrically prominent left axillary lymph nodes (Figures 3A-3C).

**Figure 4:**
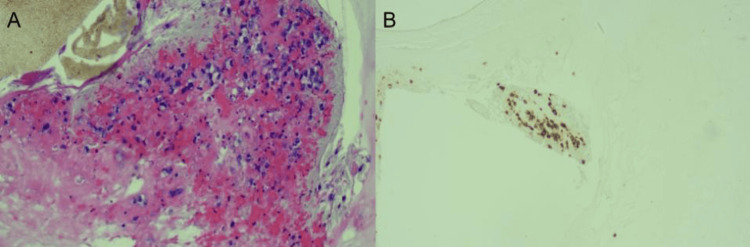
(A) Fluid cytology (hematoxylin and eosin stain) of peri-implant seroma demonstrating pleomorphic, abnormal cells with atypical nuclei. (B) Fluid cytology (immunohistochemical stain) of peri-implant seroma demonstrating strong positivity of CD30 cells.

The patient underwent aspiration of the peri-implant fluid collection. Cytology revealed atypical CD30+ cells, which were negative for CD20, CD3, and anaplastic lymphoma kinase (ALK) (Figures 4A, 4B). These findings were highly suspicious for BIA-ALCL. Few cells stained positive for CD4 and AE1/3. A core biopsy of the left axillary lymph node was benign.

**Figure 5:**
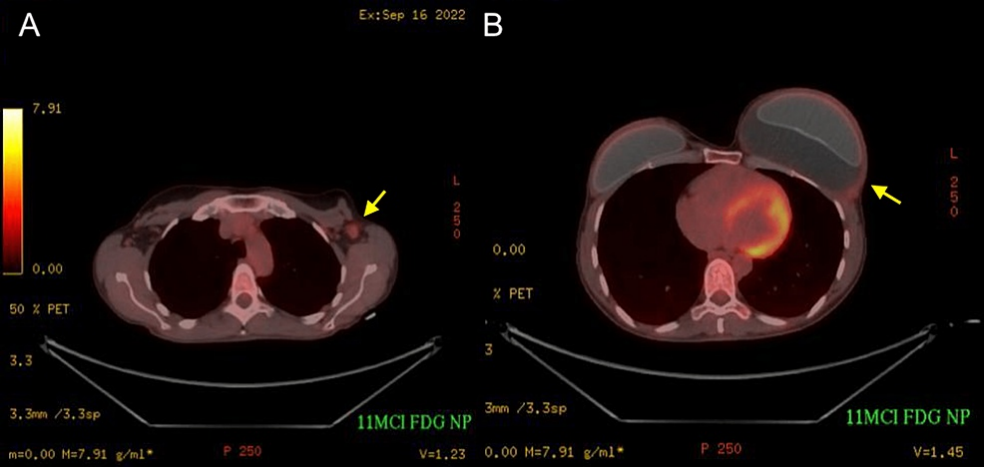
(A) Axial fluorodeoxyglucose positron emission tomography-computed tomography image demonstrating FDG-avid enlarged left axillary lymph node. (B) Axial FDG positron emission tomography-computed tomography image demonstrating only minimal fluorodeoxyglucose avidity of the peri-implant collection capsule wall.

The patient subsequently underwent preoperative positron emission tomography-computed tomography (PET-CT) imaging to determine the extent of the disease which showed no evidence of distant disease (Figures 5A, 5B).

**Figure 6:**
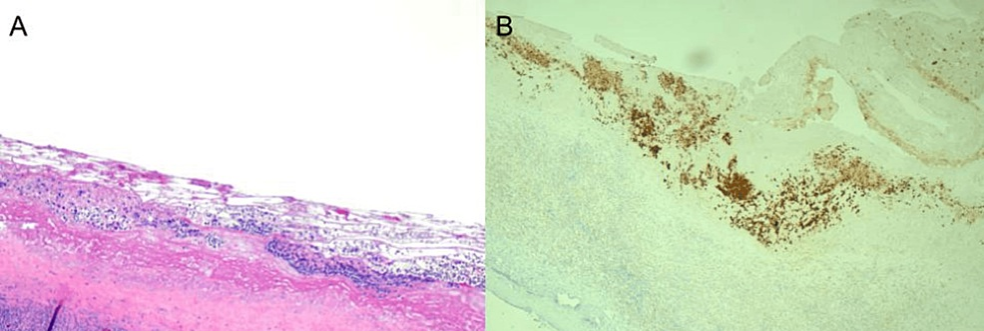
(A) Hematoxylin and eosin stain of the surgical specimen demonstrating large, scattered cells with atypical nuclei. (B) Immunohistochemistry of the surgical specimen demonstrating strong positivity of CD30.

The patient underwent bilateral capsulectomy with implant removal and left axillary lymph node excisional biopsy. The specimen from the left breast was removed intact and en bloc so that the capsule with seroma and implant was not disrupted. Surgical pathology revealed an intact fibrous pseudo-capsule with a contained seroma and a prominent reactive chronic inflammatory infiltrate. Neoplastic cells were immunoreactive for CD30, EMA, TIA1, and CD4; weakly reactive for CD2; and very weakly reactive for CD43 (Figures 6A, 6B). Stains for ALK1, CD3, CD20, PAX5, CD79a, CD8, and EBER were negative. This pattern of staining further confirmed the diagnosis of BIA-ALCL. The neoplastic cells were confined to the fibrinous lining of the pseudo-capsule and were not identified within the inflammatory infiltrate in the fibrous capsule, nor were they seen at the margins of the specimen. Left axillary lymph nodes showed reactive hyperplasia, with negative staining for CD30 and no diagnostic features of malignancy. Due to the local stage of her disease, being only stage 1, additional adjuvant therapy was not recommended.

## Discussion

BIA-ALCL is a rare CD30-positive, kinase-negative, T-cell non-Hodgkin lymphoma of the breast, affecting one in every 2,969 women with breast implants and one in 355 patients with textured implants after breast reconstruction [7]. Patients typically present an average of eight years after implant placement, with the most common presentation being a new, non-resolving peri-implant seroma [6]. The most commonly accepted hypothesis as to the pathogenesis of BIA-ALCL relates to the surface area of textured implants allowing for increased bacterial adhesion and biofilm formation which subsequently produces a chronic inflammatory state that promotes malignant lymphocyte transformation [6].

Diagnostic workup of BIA-ALCL most commonly begins with an ultrasound examination to evaluate for the presence of seroma [8]. Additionally, ultrasound has a high sensitivity for detecting associated masses, which are negative prognosticators [8]. Breast MRI with and without contrast is frequently utilized if ultrasound results are equivocal and are indicated to evaluate for capsule enhancement and the presence of solid components [8]. MRI is also a particularly useful prognostic tool, as it may detect smaller peri-implant masses/nodularity not evident with ultrasound and can determine the extent of the disease as it images the entire breast parenchyma and axillae [8]. PET-CT can be utilized to further evaluate the distant spread of disease for prognosis and perioperative workup [8].

The fluid aspirate surrounding the breast implant is required to differentiate a benign versus malignant seroma. If a mass is also present, a needle biopsy can be performed to assist workup. Pathology/cytology may reveal large, polymorphic cells with irregular nuclei, described as “horseshoe-shaped” or “kidney-shaped.” Immunohistochemistry analysis would reveal strong, positive expression of CD30 and a lack of expression of ALK [9]. Other T-cell antigens commonly associated with BIA-ALCL include CD2, CD3, CD4, CD43, and CD45 [9].

Treatment for BIA-ALCL depends upon its TNM staging, as opposed to the Ann-Arbor staging typically used for non-Hodgkin Lymphomas, as it behaves most similarly to solid tumors [9]. In general, BIA-ALCL contained within the capsule is treated with surgical resection alone, such as in this patient, while more advanced disease is combined with chemotherapy and radiation [10]. The prognosis of BIA-ALCL is promising, as it has an approximately 90% five-year survival rate [10]. However, for the minority of patients who also present with a breast mass, unlike our patient, outcomes are less favorable and have an increased rate of death [11].

As there is limited literature on BIA-ALCL in the pregnancy and postpartum period, diagnostic and management guidelines on treating BIA-ALCL during these time periods are yet to be established. However, based on the literature and our patient’s case, diagnosis and management appears to follow traditional guidelines. In the first case report published on BIA-ALCL discovered during pregnancy, the patient underwent a similar workup to the patient presented in our report, aside from not undergoing mammography imaging, likely to avoid radiation exposure during pregnancy [12]. The patient was able to carry the pregnancy to term with no fetal complications [12]. Bilateral mastectomy, which was curative for the patient’s disease, was deferred until delivery, which did not affect the patient’s prognosis [12]. While these isolated cases provide preliminary insights, it is essential to acknowledge the unique nature of each situation. As BIA-ALCL management in pregnancy and the postpartum period necessitates a delicate balance between maternal health and fetal/child well-being, further investigations encompassing larger cohorts are imperative to elucidate optimal approaches.

## Conclusions

We present a case in which a postpartum woman presented with new onset of unilateral breast swelling in the setting of having textured breast implants placed for breast augmentation seven years prior. While a rare disease that was only recently described, it is important to keep the diagnosis of BIA-ALCL in the differential diagnosis for any woman with breast implants presenting with a non-resolving breast seroma. It is also essential to acknowledge BIA-ALCL as a potential differential diagnosis for women with breast implants during the postpartum period who present with breast swelling, as breast-related concerns during this time are frequently attributed to lactation. BIA-ALCL classically presents as a pericapsular effusion in women with textured breast implants. Ultrasound is the first-line imaging modality utilized to visualize the fluid collection. Aspiration of the peri-implant fluid is used to establish diagnosis, and MRI of the breasts aids in determining the extent of disease as well as serves as a prognostic indicator. The prognosis for BIA-ALCL is excellent, especially when the disease is localized.
